# Reply to Ceccarelli et al., “At the bottom of the Pandora’s box: preserving AMR surveillance in Gaza’s collapse”

**DOI:** 10.1128/msystems.01293-25

**Published:** 2026-01-30

**Authors:** Ramya Kumar, Zaina Alqudwa, Jade Pagkas-Bather, Osama Tanous

**Affiliations:** 1 University of Chicago, Pritzker School of Medicine 12246, Chicago, Illinois, USA; 2 Al Quds University Abu-Deis - Al Azhar University branch – Gaza 361485 , Gaza, Palestine; 3 Department of Infectious Disease, University of Chicago Medicine 21727, Chicago, Illinois, USA; 4 FXB Center for Health and Human Rights, Harvard University, Boston, Massachusetts, USA; The University of Maine, Orono, Maine, USA

## REPLY

We agree with Ceccarelli et al.’s assessment of the state of infectious disease monitoring and antimicrobial stewardship in Gaza. Amidst the destruction of the healthcare system and assault on Gaza, we would like to highlight specific examples that demonstrate the severity of infectious disease in the Gaza Strip. Physicians and healthcare workers in Gaza are reporting a rapid and fatal spread of infectious illnesses and their complications demonstrated by the rise in Guillain-Barré syndrome (GBS), the reappearance of diseases, such as polio, previously eradicated with comprehensive vaccination programs, as well as a significant rise in skin infections directly attributed to the severe lack of hygiene and sanitation supplies facing hospitals and the entire population (1).

GBS is an autoimmune disease of the nervous system often caused by prolonged viral and bacterial illnesses (2). GBS is a rare disorder most often following infections from *Campylobacter jejuni*, Epstein-Barr virus, cytomegalovirus, and an immunocompromised state (3, 4). Since the start of the assault on Gaza, between June 2025 and October 2025, physicians have reported 140 suspected cases (5). The outbreak of GBS in Gaza demonstrates a multifaceted attack on the overall health of the population. Overall, food shortages and subsequent malnutrition have led to a state of exacerbated vulnerability to the progression of infectious diseases (6). Additionally, *C. jejuni*, a major risk factor for GBS, is a bacteria that can be spread through consumption of water contaminated with human or animal feces (7). While the lack of adequate testing supplies creates difficulty in identifying the causative agent of GBS, the collapse of WASH systems and the lack of portable water promote the spread of GBS-causing microbes and viruses (6). Symptoms of the disease include acute paralysis, rendering patients unable to stand and walk, eventually progressing to paralysis of the respiratory muscles, causing respiratory failure and death (8). Increase in GBS cases demonstrates the population’s lack of resources to halt fatal progression and complications of infectious diseases.

Healthcare workers in Gaza have also reported a rapid rise in skin infections during the war. Particularly, bullous and non-bullous impetigo has been prevalent among many patients in Gaza (9). Impetigo is an infectious skin rash primarily caused by Group A *Streptococcus* and *Staphylococcus aureus*, both of which can be easily treated with antibiotics (10). However, due to the blockade and resulting shortage of antibiotics, healthcare workers have had difficulty treating impetigo (11). Additionally, clinicians are reporting the spread of the rash is more severe, blistering, and widespread (11). Parasitic infections have become widespread as well (12, 13). In Gaza, scabies and lice are spreading in an endless cycle throughout the population (12, 13). Proper treatment has been challenging (12, 13). Families are unable to receive treatment due to the difficulty of maintaining hygiene in overcrowded shelters and tents that are housing 90% of the population in Gaza (14). Fungal infections have also increased the burden of infectious diseases among Gaza’s population (11). Cutaneous candidiasis and other fungal skin infections have been reported in Gaza in the setting of prolonged moisture, unwashed clothing, and poor shelter ventilation (15).

The healthcare system in Gaza has been destroyed by Israel, leaving no access to proper medicines or specialists (16). There are no infectious disease clinics or hospitals for these infections (16). Patients and healthcare workers are attempting to manage infections to the best of their ability (16, 17). However, the blockade of supplies, unprecedented escalation in violence, and overall destruction of the healthcare system have created innumerable barriers to health (17). These bacterial, parasitic, and fungal infections demonstrate one aspect of the harsh reality of life in Gaza, where even the mildest infection becomes exceptionally difficult to treat.
